# Photon‐Counting CT for Diagnosing Vertical Root Fractures in Teeth With Metal Posts: An Ex Vivo Comparative Analysis With Four CBCT Devices

**DOI:** 10.1111/iej.70095

**Published:** 2026-01-09

**Authors:** Renata M. S. Leal, Fernanda B. Fagundes, Maria F. S. A. Bortoletto, Samuel C. Kluthcovsky, Walter Coudyzer, Bruno C. Cavenago, Reinhilde Jacobs, Rocharles Cavalcante Fontenele

**Affiliations:** ^1^ Department of Restorative Dentistry Federal University of Paraná Paraná Brazil; ^2^ Department of Oral Diagnosis, Area of Dental Radiology, Piracicaba Dental School State University of Campinas (UNICAMP) São Paulo Brazil; ^3^ Department of Mechanical Engineering Federal University of Paraná Paraná Brazil; ^4^ Department of Radiology University Hospitals Leuven Leuven Belgium; ^5^ OMFS IMPATH Research Group, Department of Imaging and Pathology, Faculty of Medicine University of Leuven Leuven Belgium; ^6^ Department of Oral and Maxillofacial Surgery University Hospitals Leuven Leuven Belgium; ^7^ Department of Dental Medicine Karolinska Institutet Stockholm Sweden; ^8^ Department of Physiology, Faculty of Dentistry, Center of Excellence in Precision Medicine and Digital Health Chulalongkorn University Bangkok Thailand; ^9^ Division of Oral Radiology, Department of Stomatology, Public Health and Forensic Dentistry, School of Dentistry of Ribeirão Preto University of São Paulo (USP) Ribeirão Preto Brazil

**Keywords:** cone‐beam computed tomography, diagnostic imaging, metals, photon‐counting computed tomography, vertical root fracture

## Abstract

**Objective:**

Photon‐counting computed tomography (PCCT) represents a major innovation in X‐ray detection technology, offering improved signal efficiency and reduced electronic noise compared with cone‐beam computed tomography (CBCT), which can enhance image quality. This study aimed to evaluate the diagnostic performance of PCCT in detecting vertical root fractures (VRF), in comparison with four CBCT devices.

**Methodology:**

Eighteen single‐rooted teeth were endodontically treated, and VRF was induced in eight of them. Each tooth was individually placed into the mandibular first premolar empty socket of an anthropomorphic phantom and scanned under three conditions: without a metal post, with a nickel‐chromium metal post (Ni‐Cr), and with a cobalt‐chromium metal post (Co‐Cr) in five CT devices: the NAEOTOM Alpha PCCT (Siemens Healthineers) device and four CBCT devices (3D Accuitomo 170—Morita, Veraview X800—Morita, NewTom VGi evo—NewTom, and Carestream 9600—Carestream). The highest‐resolution protocol available on each device was used, resulting in a total of 270 scans. Five experienced dentomaxillofacial radiologists independently and blindly evaluated the scans using a five‐point confidence scale. Diagnostic accuracy was assessed by calculating the area under the ROC curve (AUC), sensitivity, and specificity, with results compared by two‐way ANOVA with post hoc Tukey's test (*α* = 0.05).

**Results:**

NewTom VGi and PCCT devices showed significantly higher AUC values than the Veraview X800, regardless of the metal post material (*p* < 0.05). CS9600 and PCCT devices exhibited significantly higher sensitivity values in diagnosing with Ni‐Cr posts than the Accuitomo 3D and Veraview X800 devices (*p* < 0.05). With the Co‐Cr metal post, the NewTom VGi, CS9600, and PCCT devices showed significantly higher sensitivity values compared to the Veraview X800 device (*p* < 0.05). There were no statistically significant differences in specificity, regardless of the CT device or metal post material (*p* > 0.05).

**Conclusions:**

The NEAOTOM Alpha PCCT showed high diagnostic accuracy for VRF detection in an ex vivo model, comparable to high‐resolution CBCT devices, highlighting its diagnostic performance under controlled ex vivo conditions.

## 1 Introduction

During endodontic treatment or retreatment, excessive removal of healthy dentine, prolonged exposure to irrigating solutions or intracanal medications, excessive forces during obturation, or inadequate final restorations can compromise the integrity of the tooth structure, potentially leading to the formation of cracks or vertical root fractures (VRF) (Adorno et al. 2013; Lertchirakarn et al. 2003; Patel et al. 2022; Shemesh et al. 2011; Silva et al. 2020). A VRF is a longitudinal fracture that involves cementum, dentine, and the root canal, typically extending along the long axis of the root towards the root apex (Freitas et al. 2019; Patel et al. 2022). Due to the superimposition of surrounding anatomical structures, periapical radiographs often fail to reveal fractures (Hassan et al. 2009; PradeepKumar et al. 2016). Clinical signs and symptoms are usually nonspecific, often resulting in an inconclusive diagnosis (Gaêta‐Araujo et al. 2017; Patel et al. 2022; Zhang et al. 2019).

Cone beam computed tomography (CBCT) is a valuable diagnostic modality for VRF, as it provides three‐dimensional imaging without anatomical overlap, thereby overcoming one of the main limitations of conventional radiographs (Liao et al. 2017; Scarfe et al. 2012). However, CBCT has notable limitations, particularly in the presence of high‐density materials. These materials, such as metal posts, can produce beam hardening artefacts that impair image quality by either obscuring or mimicking fracture lines (Gulibire et al. 2020; Zhang et al. 2019). These artefacts typically appear as hyperdense streaks or hypodense bands and result from the differential attenuation of the X‐ray beam as it passes through high‐density materials (Chang et al. 2016; Fontenele et al. 2018; Queiroz et al. 2018). Despite these limitations, high‐resolution CBCT remains the clinical imaging modality with the highest diagnostic accuracy for VRF (Salineiro et al. 2017). Even when the fracture line itself is not clearly visible, CBCT enables the identification of characteristic patterns of periradicular bone loss that are considered pathognomonic for VRF (Arkhipova et al. 2024; Byakova et al. 2019; Patel et al. 2022).

Photon‐counting computed tomography (PCCT), introduced to the market in 2021, uses a narrow, fan‐shaped X‐ray beam similar to that of conventional CT, whereas CBCT employs a wider cone‐ or pyramid‐shaped beam. The major technological advancement of PCCT lies in its detector design: instead of conventional energy‐integrating detectors that rely on a scintillator layer to convert X‐ray photons into visible light, PCCT uses semiconductor diode sensors arranged in a single layer. Each absorbed photon directly generates an electrical charge cloud that is individually collected under an applied bias voltage, eliminating the intermediate light‐conversion step (Al‐Haj Husain et al. 2025; Fontenele et al. 2023; Tortora et al. 2022; Willemink et al. 2018). The absence of scintillator material and reflective lamellae allows for smaller detector pixels and reduced optical cross‐talk, which may contribute to improved spatial resolution.

PCCT achieves spatial resolution of approximately 200 μm, approaching that of high‐resolution CBCT systems (80–125 μm), while offering improved contrast resolution, higher signal‐to‐noise ratio, and reduced electronic noise. Additional advantages include the potential for lower radiation doses compared to multidetector CT, shorter scan times, and high‐resolution image reconstruction. PCCT also provides superior contrast differentiation between hard and soft tissues, a notable improvement over CBCT (Al‐Haj Husain et al. 2025; Fontenele et al. 2023; Tortora et al. 2022; Willemink et al. 2018). Previous studies have shown that PCCT outperforms high‐resolution CBCT in dental diagnostic tasks (Ruetters et al. 2022; Vanden Broeke et al. 2021), including the detection of fine endodontic structures (Fontenele et al. 2023). However, despite its excellent performance, no scientific evidence currently supports the use of PCCT for diagnosing VRF in teeth restored with metal posts, a scenario particularly susceptible to severe artefacts.

Therefore, this study aimed to assess the diagnostic accuracy of PCCT for detecting VRF in teeth with intracanal metal posts and to compare it with four different CBCT devices. The null hypothesis was that PCCT and the CBCT scanners would not differ in their ability to diagnose VRF in this context.

## 2 Material and Methods

**FIGURE 1:**
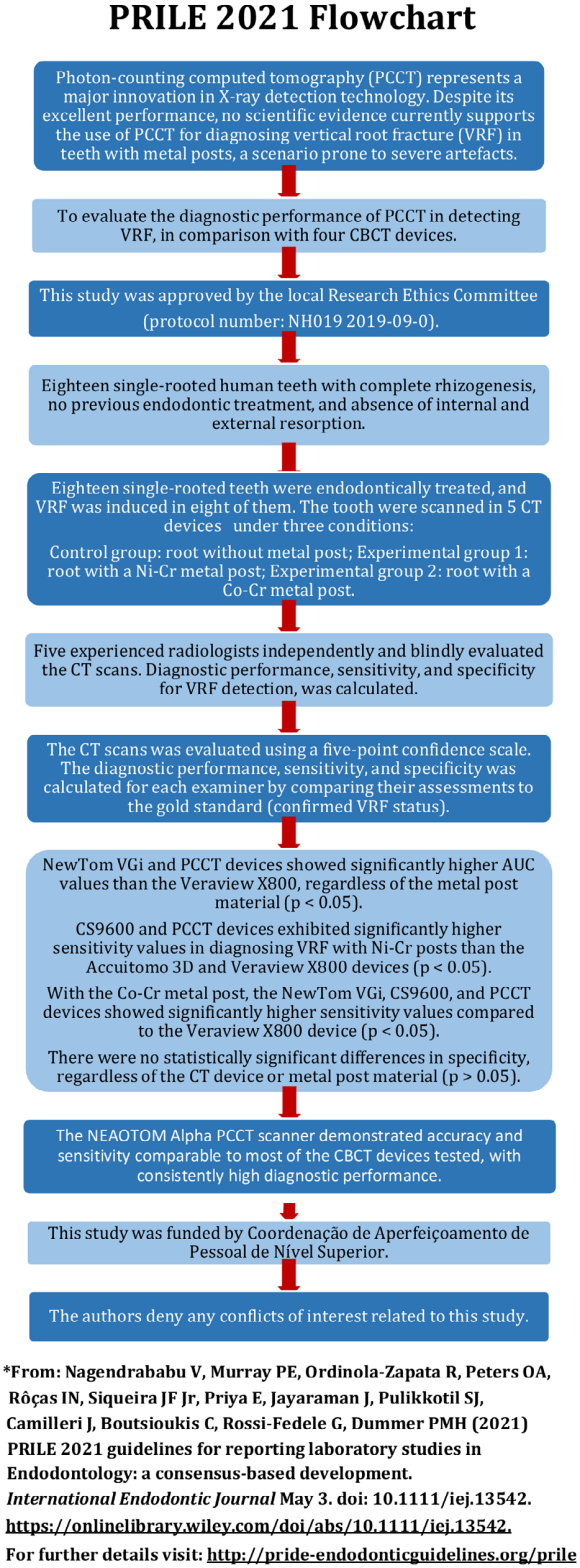
PRILE flowchart.

This study was approved by the Local Ethics Committee (protocol number: NH019 2019‐09‐03) and was conducted in accordance with the World Medical Association's Declaration of Helsinki on medical research, adhering to the Preferred Reporting Items for Laboratory studies in Endodontology (PRILE) 2021 guidelines (Figure 1). A human skull and a dentate dry adult human mandible, covered with Mix D validated for this study setup, were used in the present study (Fontenele et al. 2023; Wanderley et al. 2022).

### 2.1 Sample Selection and Preparation

Eighteen single‐rooted human teeth, freshly extracted due to periodontal disease, were selected according to the following inclusion criteria: complete rhizogenesis, no previous endodontic treatment, and absence of internal and external resorption. Teeth were cleaned and rinsed with saline solution, then stored in a 0.1% thymol solution for up to 30 days prior to the experiment. Two different intrarradicular metal posts were prepared: one Nickel‐Chromium (Ni‐Cr) and one Cobalt‐Chromium (Co‐Cr).

The crowns of the teeth were sectioned at the cemento‐enamel junction using a diamond disc mounted on an Isomet low‐speed precision cutter (Isomet 1000, Buehler Ltd., Lake Bluff, USA) to eliminate potential enamel‐induced artefacts. All root canals were endodontically prepared using a size 40.06 diameter and taper instrument (Reciproc Blue, VDW, Munich, Germany) operated in reciproc motion in an X‐Smart Plus motor (Dentsply Sirona, Ballaigues, Switzerland), up to the working length (1 mm short of the apical foramen). During instrumentation, root canals were irrigated with a 2.5% sodium hypochlorite (NaOCl) solution (Asfer, São Caetano do Sul, Brazil), using a 5 mL syringe (BD, Curitiba, Brazil) and a 29G NaviTip needle (Ultradent Inc.; South Jordan, UT, USA). Then, two‐thirds of each root canal were prepared for post placement using a Largo number #2 drill (Dentsply Maillefer, Ballaigues, Switzerland) attached to a low‐speed contra‐angle.

**FIGURE 2:**
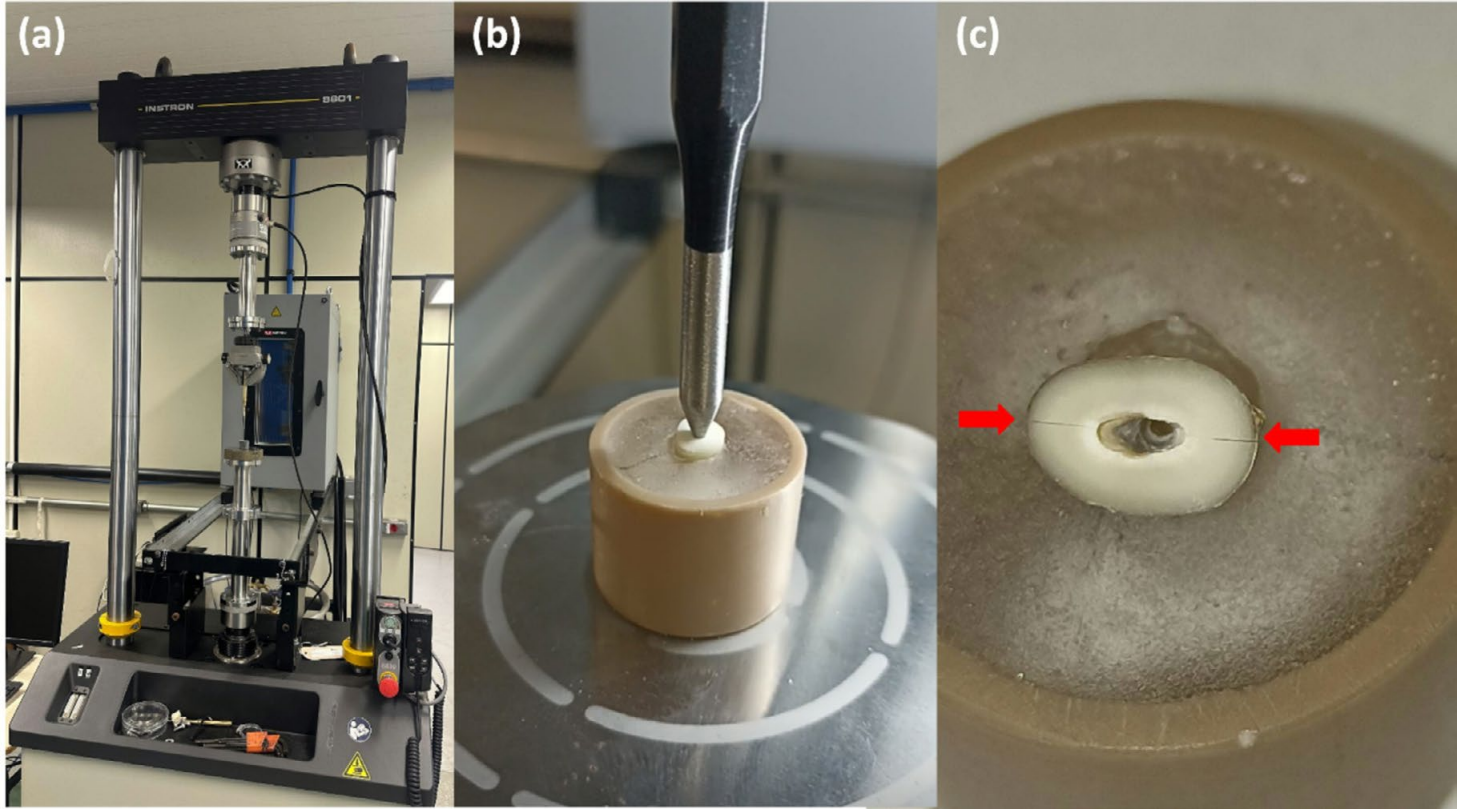
Induction of vertical root fracture: (a) Instron 8802 machine (a); (b) Conic metal tip inserted into the root canal opening; (c), Red arrows indicating the presence of VRF.

The sample was divided into two groups: an experimental group (8 teeth) and a control group (10 teeth). The eight teeth in the experimental group were temporarily fixed in acrylic resin blocks. VRF were then induced using an Instron 8802 machine (Instron Corporation, Canton, MA, USA), as shown in Figure 2a. A conical metal tip was inserted into the root canal opening, and force was applied until fracture occurred, at which point the machine stopped automatically (Figure 2b), effectively replicating the natural process of root fracture initiation. This method enables the generation of fractures that closely resemble clinical conditions. The roots were then removed from the resin blocks, and the presence of a VRF was confirmed by transillumination and visual inspection (Figure 2c).

**FIGURE 3:**
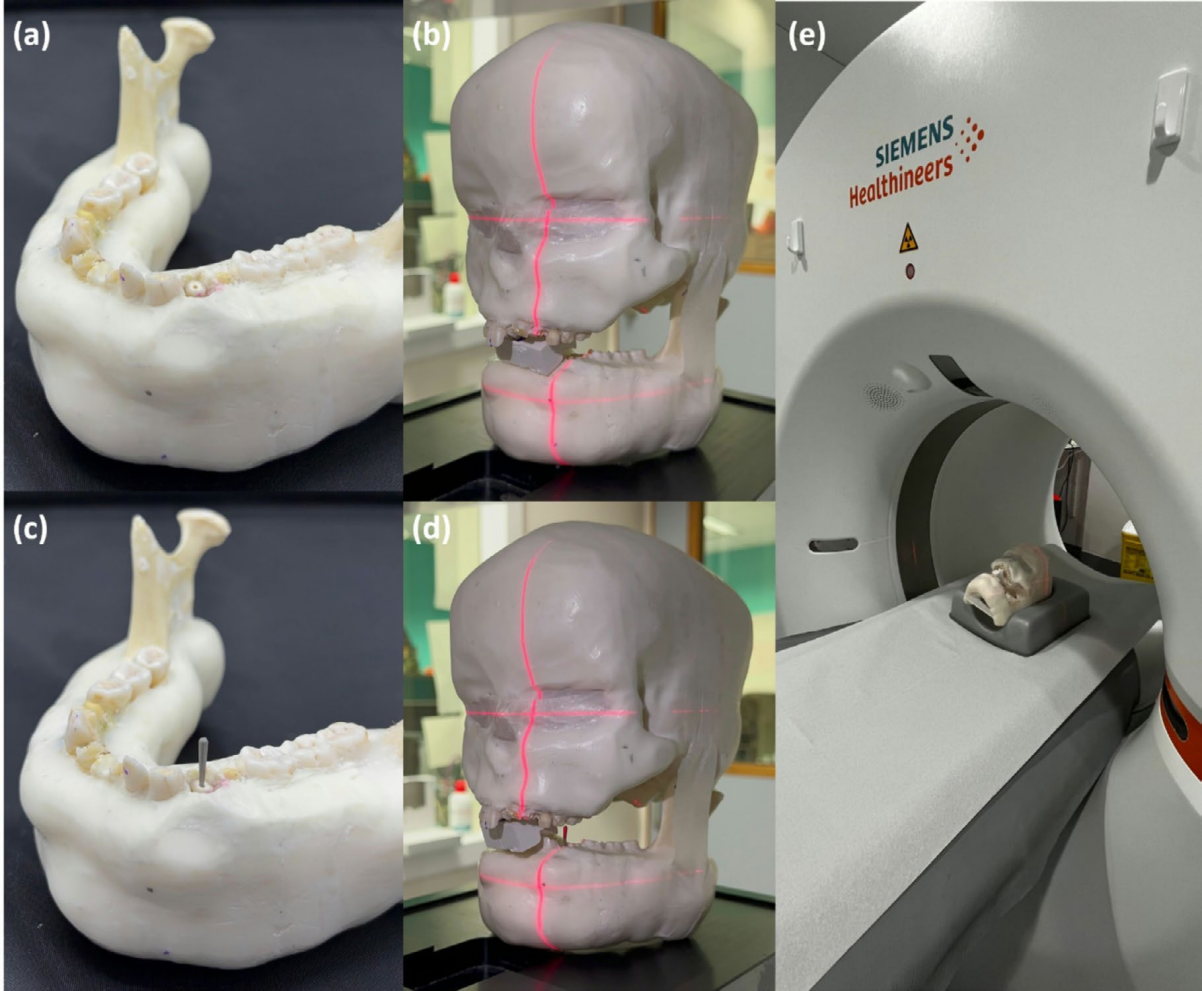
Dentate dry adult human mandible covered with Mix D with a root individually positioned in a mandibular first premolar edentulous alveolus in the absence (a) and presence of intracanal metal post (c). The phantom with a root individually positioned in a mandibular first premolar edentulous alveolus, positioned in a standardised position, using the reference guide lines from the CBCT device, in the absence (b) and presence of intracanal metal post (d). The phantom positioned in a standardised position, using the reference guide lines from PCCT NAEOTOM Alpha device (e).

The roots from both the control and experimental groups were individually positioned in the edentulous alveolus of a mandibular first premolar within a custom‐made phantom. This phantom consisted of a human skull and a dentate dry adult human mandible covered with Mix D, a material that simulates the absorption and scattering of X‐rays in soft tissues (Wanderley et al. 2022; Fontenele et al. 2023). The roots were scanned both with and without the presence of intraradicular metal posts made of Ni‐Cr and Co‐Cr alloys (Figure 3).

### 2.2 Image Acquisition

The phantom was positioned using the reference positioning lights of the CBCT and PCCT devices (Figure 3). The scout tool was then used to centre the tooth within the field of view (FOV). The imaging devices and acquisition parameters are detailed in Table 1.

**TABLE 1:** Scanning parameters for adults of the different imaging devices.

Devices	Parameters					
	FOV (cm)	Voxel (mm)	Tube voltage (kVp)	Tube current (mA)	Time of exposure (s)	DAP—Dose area product (mGy/cm^2^)
3D Accuitomo 170	4 × 4	0.08	90	7.5	30.8	10.6
Veraview X800	4 × 4	0.08	100	7	17.9	10.98
Newtom VGI evo	5 × 5	0.125	110	7.48	6	4.13
Carestream 9600	5 × 5	0.075	120	6.3	8.5	6.32
Photon‐counting CT (NEAOTOM Alpha)	8 × 8	0.2	120	15	3.84	238.3

Three sets of images were acquired per tooth under the following conditions:

- Control group: root without metal post;
- Experimental group 1: root with a Ni‐Cr metal post;
- Experimental group 2: root with a Co‐Cr metal post.

**FIGURE 4:**
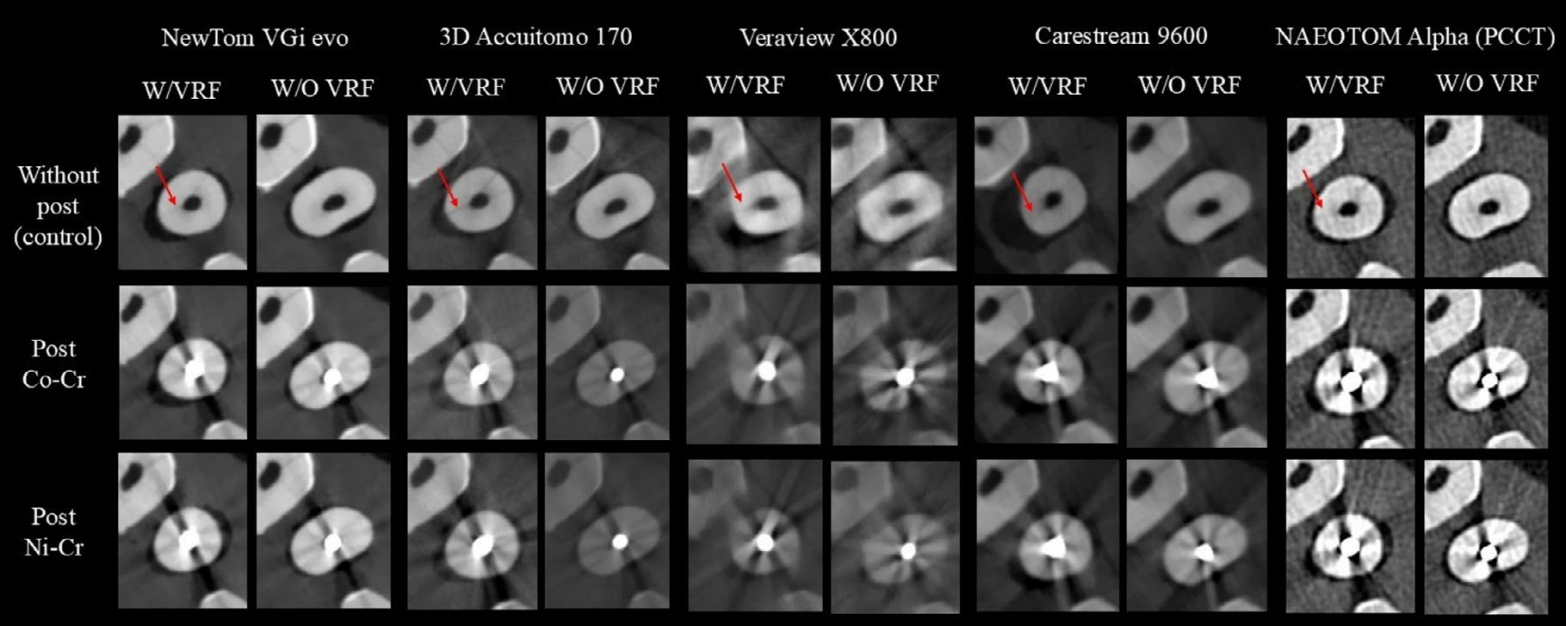
Axial slices illustrating the presence or absence of vertical root fracture across all CBCT and PCCT devices under different conditions (control, post‐Co‐Cr, and post‐Ni‐Cr). Arrows indicate the VRF. VRF, vertical root fracture; W/, with; W/O, without.

In total, 270 CT scans were acquired (18 teeth × 5 CT devices × 3 conditions). Figure 4 represents the axial reconstructions of the different groups and devices assessed.

### 2.3 Image Evaluation

CT scans were exported in Digital Imaging Communications in Medicine (DICOM) format and randomised. Five oral and maxillofacial radiologists, each with more than 4 years of experience, independently evaluated all the scans. The examiners were blinded to the study variables and were calibrated prior to the assessment. Each scan was evaluated for the presence of VRF using a 5‐point scale: 1—absence of VRF; 2—probable absence of VRF; 3—uncertainty; 4—probable presence of VRF; and 5—presence of VRF. RadiAnt DICOM Viewer (Medixant, Poznań, Poland) was chosen as the DICOM viewer because it can visualise scans from all of the CBCT and PCCT devices tested in this study, and it provides standardised image display parameters. Using this software allowed all examiners to conduct an unbiased and blinded evaluation because the interface does not display information about the imaging device or acquisition settings. This minimises potential interpretation bias related to the imaging scanners under investigation. To minimise visual fatigue, examiners were instructed to assess a maximum of 10 scans per day. Post‐processing adjustments (e.g., contrast, brightness, and zoom adjustments) were allowed. One month after the initial evaluation, 30% of the sample (*n* = 81 scans) was randomly selected and re‐evaluated by the examiners to assess intra‐ and inter‐examiner agreements.

### 2.4 Statistical Analysis

Intra‐ and interobserver agreements were evaluated using the weighted Kappa index and interpreted according to the Landis and Koch scale (Landis and Koch 1977). Diagnostic performance, including the area under the receiver operating characteristic curve (AUC), sensitivity, and specificity for VRF detection, was calculated for each examiner by comparing their assessments to the gold standard (confirmed VRF status). Each of the five evaluators analysed the same set of images under all experimental conditions (i.e., five devices and three metal post experimental groups). For each evaluator and condition, the performance metrics were calculated based on all image classifications, and the evaluator was considered the experimental unit. Therefore, the diagnostic performance was compared using a two‐way ANOVA with the type of CT device and metal post experimental group as factors, followed by Tukey's post hoc test for multiple comparisons. All statistical analyses were performed using MedCalc Statistical Software (version 20.116; MedCalc Software Ltd., Ostend, Belgium), with a significance level of 5% (*α* = 0.05).

A post hoc statistical power was estimated from the observed effect size (partial η^2^) obtained in the two‐way ANOVA. The partial η^2^ was converted to Cohen's f according to the equation $f=\sqrt{\left(\eta_{p}^{2}/1-\eta_{p}^{2}\right)}$. The achieved power (1 − *β*) was then calculated using an *α* level of 0.05, based on the total sample size and the number of groups, employing the FTestAnovaPower function from the statsmodels package (Python Software Foundation). The resulting statistical power was 90%, indicating an adequate sensitivity to detect the observed effect size.

## 3 Results

Interexaminer agreement ranged from slight (0.34) to substantial (0.70) and intra‐examiner agreement ranged from moderate (0.45) to substantial (0.78) (Table 2).

**TABLE 2:** Intra‐ and interobserver agreements for the detection of vertical root fracture.

Examiner	1	2	3	4	5
1	0.51	0.53	0.51	0.51	0.36
2		0.58	0.42	0.70	0.34
3			0.45	0.44	0.53
4				0.61	0.38
5					0.78

The AUC values are presented in Table 3. Regardless of the metal post material used, the NewTom VGi and PCCT devices showed statistically significantly higher AUC values compared to the Veraview X800 device (*p* < 0.05). The 3D Accuitomo and CS9600 devices demonstrated intermediate performance, with no statistically significant differences compared to the other CBCT devices tested (*p* > 0.05).

**TABLE 3:** Mean (standard deviation) of area under the ROC curve (AUC) values by experimental condition, according to metal post material and imaging device.

Metal post material	Imaging device				
	3D Accuitomo 170^AB^	Veraview X800^B^	NewTom VGi^A^	CS9600^AB^	PCCT^A^
Control	0.73 (0.10)	0.53 (0.28)	0.76 (0.16)	0.70 (0.18)	0.76 (0.18)
Ni‐Cr	0.52 (0.08)	0.48 (0.08)	0.71 (0.12)	0.67 (0.30)	0.64 (0.31)
Co‐Cr	0.60 (0.13)	0.51 (0.09)	0.70 (0.13)	0.65 (0.22)	0.70 (0.23)

*Note:* Different uppercase letters indicate statistically significant differences among the devices, regardless of the type of metal post material (*p* < 0.05).

Table 4 presents the sensitivity values among the different devices for the control, Ni‐Cr, and Co‐Cr groups. No statistically significant differences in sensitivity were observed among the devices for VRF diagnosis in the control group (*p* > 0.05). In the Ni‐Cr metal post group, the CS9600 and PCCT devices demonstrated significantly higher sensitivity compared to the 3D Accuitomo and Veraview X800 (*p* < 0.05). The NewTom VGi showed intermediate sensitivity, which was not significantly different from that of the 3D Accuitomo, CS9600, and PCCT (*p* > 0.05). However, its sensitivity was statistically significantly higher than that of the Veraview X800 (*p* > 0.05). For the Co‐Cr metal post group, the NewTom VGi, CS9600, and PCCT all showed statistically significant higher sensitivity than the Veraview X800 (*p* < 0.05). The 3D Accuitomo demonstrated intermediate sensitivity, with no statistically significant differences compared to the other CBCT devices (*p* > 0.05).

**TABLE 4:** Mean (standard deviation) of sensitivity values by experimental condition, according to metal post material and imaging device.

Metal post material	Imaging device				
	3D Accuitomo 170	Veraview X800	NewTom VGi	CS9600	PCCT
Control	0.67 (0.07)^Aa^	0.47 (0.25)^Aa^	0.75 (0.23)^Aa^	0.47 (0.25)^Aa^	0.75 (0.23)^Aa^
Ni‐Cr	0.22 (0.16)^BCb^	0.10 (0.10)^Cb^	0.62 (0.09)^ABa^	0.72 (0.35)^Aa^	0.65 (0.27)^Aa^
Co‐Cr	0.40 (0.24)^ABa^	0.10 (0.10)^Bb^	0.62 (0.09)^Aa^	0.67 (0.36)^Aa^	0.57 (0.30)^Aa^

*Note:* Different uppercase letters indicate statistically significant differences among the imaging devices within each metal post material group (*p* < 0.05). Different lowercase letters indicate statistically significant differences among the metal post materials within each imaging device (*p* < 0.05).

When comparing the different groups (control, Ni‐Cr, and Co‐Cr) within each device, the 3D Accuitomo showed significantly higher sensitivity values in the control and Co‐Cr groups compared to the Ni‐Cr group (*p* < 0.05). In the Veraview X800 device, the control group also exhibited significantly higher sensitivity than both the Ni‐Cr and Co‐Cr groups (*p* < 0.05). No significant differences in sensitivity were observed among the three groups when using the NewTom VGi, CS9600, or PCCT devices (*p* > 0.05).

Table 5 presents the specificity values for the Control, Ni‐Cr, and Co‐Cr groups across the different devices. No statistically significant differences in specificity were observed among the devices and the groups evaluated (*p* > 0.05).

**TABLE 5:** Mean (standard deviation) of specificity values by experimental condition, according to metal post material and imaging device.

Metal post material	Imaging device				
	3D Accuitomo 170	Veraview X800	NewTom VGi	CS9600	PCCT
Control	0.74 (0.27)	0.60 (0.38)	0.72 (0.30)	0.74 (0.24)	0.70 (0.30)
Ni‐Cr	0.80 (0.10)	0.86 (0.11)	0.78 (0.20)	0.62 (0.33)	0.66 (0.36)
Co‐Cr	0.80 (0.12)	0.92 (0.08)	0.70 (0.21)	0.60 (0.31)	0.82 (0.20)

*Note:* There was no statistically significant influence on specificity results, regardless of the metal post material or the imaging device tested (*p* > 0.05).

## 4 Discussion

The present study provides valuable insights into the diagnostic performance of the PCCT NEAOTOM Alpha device for VRF detection in the presence of metal posts, compared to four CBCT devices. The PCCT NEAOTOM Alpha device demonstrated diagnostically acceptable levels of accuracy and sensitivity in detecting VRF in the presence of metal posts. Its performance was comparable to that of the 3D Accuitomo 170, NewTom VGi evo, and CS9600 CT scanners, and significantly superior to that of the Veraview X800. These findings support the confirmation of the null hypothesis, indicating that PCCT imaging can be reliably used for the assessment and diagnosis of VRF under controlled ex vivo conditions.

Artefacts produced by metal posts in CBCT images negatively impact the diagnosis of VRF (Gaêta‐Araujo et al. 2017; Gaêta‐Araujo et al. 2020; Lagos de Melo et al. 2023; Ruiz et al. 2024). Recent studies have highlighted the artefact reduction capabilities of the PCCT device (Vanden Broeke et al. 2021; Zanon et al. 2024). It has been shown to produce images with fewer artefacts in the presence of high‐atomic‐number materials (e.g., dental implants) when compared to conventional CBCT devices (Al‐Haj Husain et al. 2025). PCCT has also been reported to outperform conventional CBCT devices in detecting apical osteolysis and fine dental structures (Ruetters et al. 2022). Differences in beam geometry and in the underlying detection principle affect key imaging parameters such as spatial resolution, scatter, and noise characteristics. These technical distinctions may therefore explain the image quality differences observed between PCCT and CBCT. The findings of our study are consistent with these reports. Compared to conventional CBCT devices, PCCT demonstrated high accuracy and sensitivity in diagnosing VRF, even in the presence of different metal posts. Furthermore, although a wide range of CBCT devices is available on the market, the CBCT devices used in this study are particularly suitable for endodontic acquisitions, as most other units tend to generate excessive artefacts in the presence of metal posts, which could render the diagnosis unreliable or even impossible, as demonstrated in previous studies (Pinto et al. 2023; Wanderley et al. 2020, 2022). To the best of our knowledge, this is the first study to evaluate a PCCT device in comparison with four high‐resolution CBCT devices dedicated to endodontic imaging.

All scans were analysed using the same DICOM viewer software, which was compatible with both conventional CBCT and PCCT images. Although a previous study reported strong performance of the Veraview X800 device in evaluating fine anatomical structures (Pinto et al. 2021), the use of non‐native software for image analysis in the present study may have contributed to the lower accuracy and sensitivity observed, particularly in the presence of metal posts. Therefore, these findings should be interpreted with caution regarding their clinical applicability, as the Veraview X800 demonstrates improved diagnostic performance when its images are evaluated using its proprietary software.

The 3D Accuitomo, which is cited in the literature as a reference for evaluating fine anatomical structures (Pinto et al. 2021; Fontenele et al. 2023), generally showed intermediate results in terms of accuracy and sensitivity for diagnosing VRF in this study. However, it is important to note that a previous study comparing the 3D Accuitomo 170 and PCCT devices for detecting fine structures did not include evaluations in the presence of a metal post, a factor that may explain the divergent results observed in our study (Fontenele et al. 2023). In addition, the previous study by Fontenele et al. (2023) evaluated the images statically, whereas in the present study, images were assessed dynamically, which more closely simulates a real clinical setting.

Overall, the NewTom VGi and CS9600 devices showed accuracy and sensitivity values for diagnosing VRF that were similar to those of PCCT. However, these findings contrast with those of a previous study, in which PCCT demonstrated superior performance compared to the NewTom VGi in detecting fine anatomical structures (Fontenele et al. 2023). It is important to note, however, that the structures evaluated in that study were not associated with the presence of metal posts, an important factor that may help explain the differences observed in our results.

The CBCT devices included in the present study were not selected based on convenience. Instead, the selection was grounded in robust prior evidence from two comprehensive investigations assessing image quality and artefact expression across multiple CBCT devices. First, in a previous study (Wanderley et al. 2022), which compared 13 CBCT devices, significant variations were observed in artefact expression caused by high‐density materials such as metallic posts. A second study (Pinto et al. 2021) conducted under comparable experimental conditions further evaluated 10 CBCT devices for their ability to visualise fine endodontic structures, including root cracks. Both studies confirmed that artefacts substantially compromise diagnostic accuracy in certain CBCT devices, particularly in endodontic applications where fine detail visualisation is essential (Wanderley et al. 2020). Therefore, only CBCT devices that demonstrated excellent or acceptable image quality in the presence of metallic posts were included in the present investigation. This criterion ensured that the comparison with PCCT was both scientifically valid and clinically meaningful, as the inclusion of lower‐performing devices would have introduced excessive artefacts and biased the analysis by artificially favouring PCCT performance.

Although no statistically significant differences in specificity were observed among the evaluated CT devices or metal post conditions, the balance between sensitivity and specificity warrants careful interpretation. Increased sensitivity improves the detection of true VRF but may also elevate the risk of false‐positive findings, which is clinically relevant given that VRF overdiagnosis may lead to unnecessary tooth extraction. In the present study, the PCCT device achieved high overall diagnostic accuracy while maintaining specificity comparable to the other systems, suggesting that improved sensitivity was not accompanied by a disproportionate increase in false‐positive diagnoses under the tested conditions. It should also be emphasised that fractures were not artificially created to match voxel dimensions, but rather to approximate realistic fracture characteristics. Coincidentally, most of the root fractures present had dimensions compatible with the 200 μm voxel size, which is consistent with the relatively high diagnostic performance observed for PCCT in the control group (i.e., without metal posts), with diagnostic metric values ranging from 0.70 to 0.76.

However, it is important to acknowledge that the root fractures artificially induced using the Instron 8802 machine were created without strict control over fracture width. Consequently, a limited number of fractures may have had dimensions below the voxel size of the PCCT acquisition, potentially rendering them undetectable by the 200 μm PCCT protocol, while still being detectable by high‐resolution CBCT devices operating with smaller voxel sizes (80–125 μm). Nevertheless, we believe that the proportion of such sub‐voxel fractures was low, as reflected by the overall good diagnostic performance observed for PCCT and the fact that some CBCT devices demonstrated lower diagnostic accuracy despite their smaller voxel sizes. This suggests that factors beyond voxel size also contribute to fracture detectability, including the intrinsic advantages of PCCT, such as reduced electronic noise, improved signal‐to‐noise and contrast‐to‐noise ratios, and enhanced edge definition (Fontenele et al. 2023; Willemink et al. 2018), which may collectively compensate for its larger voxel size and support reliable fracture detection.

The differences observed among the evaluated devices may also be attributed to their distinct image acquisition and post‐processing characteristics. In this sense, the superior performance of PCCT in detecting VRF can be explained by its technical features, including increased contrast resolution, high signal‐to‐noise ratio, fast scanning speed, and small voxel size, all of which contribute to enhanced image resolution (Fontenele et al. 2023; Pinto et al. 2021). Notably, as PCCT is a hospital‐based imaging modality, head and neck examinations are frequently requested by medical doctors in polytraumatized patients with head and neck injuries. In such cases, traumatic events may involve the dentoalveolar region, resulting in dental fractures, including root fractures. This clinical context raises the question of whether PCCT examinations acquired for other medical diagnostic purposes, when encompassing the maxillofacial region and dental arches within the field of view, may also enable incidental dentomaxillofacial assessment. In this case, the acquired images could allow for incidental evaluation of VRF, which could avoid the need for an additional CBCT scan and its associated radiation exposure.

It is important to note that these results do not suggest using PCCT as a substitute for conventional CBCT imaging, as its radiation dose level remains higher. In addition to the high cost, limited availability, and lack of standardised protocols, it should also be noted that, in PCCT, a VRF must have a width of at least 0.2 mm to be visualised, whereas in CBCT, fractures of even smaller dimensions can be detected. Additionally, most conventional CBCT devices demonstrated similar accuracy and sensitivity values for diagnosing VRF. Therefore, PCCT should be regarded as a reliable complementary tool for the diagnosis of VRF only when a PCCT scan has already been obtained for other clinical indications, and the evaluation of a potential VRF represents a secondary objective. This cautious interpretation aligns with ethical principles of radiation protection, avoiding additional patient exposure solely for diagnostic verification. These results may also encourage further technological advancements, guiding manufacturers to incorporate PCCT principles into future CBCT system designs with the aim of enhancing diagnostic accuracy and image quality. Moreover, the findings from this investigation highlight the need for future optimization studies focused on adjusting acquisition parameters to reduce patient radiation dose while maintaining or improving image quality and diagnostic performance across various dental diagnostic tasks, such as the detection of root fractures.

The main strengths of this study include the use of natural teeth and the use of an anthropomorphic phantom covered with Mix‐D, which simulates X‐ray attenuation by the patient's soft tissues. This approach better reflects clinical conditions and avoids reliance on artificial simulations, such as mandibles immersed in water containers (Ruiz et al. 2024). However, as a limitation inherent to an ex vivo design, it is important to note that clinical predictors of VRF, such as thickening of the periodontal ligament, bone defects, and clinical signs and symptoms, which are essential for final diagnosis, were not reproduced (Patel et al. 2022). This factor is also reflected in the moderate values of inter‐examiner agreement (Landis and Koch 1977), since the diagnosis of VRF is already challenging in clinical scenarios, particularly in the presence of intracanal metal posts, and becomes even more difficult without these clinical predictors.

Despite these limitations, ex vivo experimental designs are well established in the literature, especially in pioneering studies involving ionising radiation‐based imaging techniques. Such approaches are necessary to ensure scientific validation of novel imaging technologies under standardised and reproducible conditions, while avoiding unnecessary radiation exposure to patients. This methodology is thus ethically justified and scientifically appropriate, as it enables controlled and sequential imaging of the same specimen, which would not be feasible in vivo. Furthermore, this design minimises variability by allowing direct comparison among post materials within identical anatomical contexts.

Another limitation of this study is the absence of motion artefacts. In real patients, artefacts resulting from muscle tremor and involuntary movements may further hinder image interpretation (Nardi et al. 2016). Additionally, this investigation evaluated a single PCCT system, reflecting the current limited availability of commercially available PCCT technology. Consequently, the findings cannot be generalised beyond the evaluated system. Further validation across different PCCT systems will be necessary as additional devices become clinically available. The absence of obturation materials (e.g., gutta‐percha and endodontic sealer) in teeth with metal posts also represents a limitation, as these materials are prone to blooming artefacts on CBCT images, potentially leading to volumetric distortions and false impressions of complete obturation or root perforation (Rodrigues et al. 2021). Nevertheless, the experimental model adopted allowed for the sequential testing of different post materials within the same tooth during scanning, thereby providing a reliable and standardised framework for assessing imaging performance across post types. Future investigations are warranted to validate these findings in clinical scenarios, including the assessment of patient‐level diagnostic outcomes and the evaluation of emerging PCCT systems, in compliance with ethical principles.

## 5 Conclusion

Under controlled ex vivo conditions, the NEAOTOM Alpha PCCT device demonstrated high diagnostic accuracy and sensitivity for VRF detection, comparable to most high‐resolution CBCT devices evaluated. Further studies assessing patient‐level outcomes are required to determine whether these findings extend beyond the technical feasibility observed.

## Author Contributions

**Renata M. S. Leal:** conceptualization, methodology, investigation, resources, experimental procedures, writing and funding acquisition. **Fernanda B. Fagundes:** methodology, software, formal analysis, experimental procedures, review, and editing. **Maria F. S. A. Bortoletto:** methodology, software, formal analysis, experimental procedures, review, and editing. **Samuel C. Kluthcovsky:** methodology, software, and experimental procedures. **Walter Coudyzer:** methodology, investigation, review, and editing. **Bruno C. Cavenago:** methodology, investigation, resources, review, and editing (supporting). **Reinhilde Jacobs:** methodology, investigation, validation, writing, review, and editing (supporting). **Rocharles Cavalcante Fontenele:** conceptualization, methodology, investigation, validation, writing, statistical analyses, supervision, review, and editing (lead).

## Funding

This study was partially funded by Coordenação de Aperfeiçoamento de Pessoal de Nível Superior (CAPES).

## Ethics Statement

This experiment was approved by the Local Ethics Committee, registered under number NH019 2019‐09‐03.

## Conflicts of Interest

The authors declare no conflicts of interest.

## Data Availability

The data that support the findings of this study are available from the corresponding author upon reasonable request.
